# Supplementary material for: Deep mutational scanning of rabies glycoprotein defines mutational constraint and antibody-escape mutations
Source: bioRxiv. 2024 Dec 17:2024.12.17.628970. Preprint. [Version 1] doi: 10.1101/2024.12.17.628970 (PMC11702696; doi:10.1101/2024.12.17.628970)
Supplement: Supplement 1 [file NIHPP2024.12.17.628970v1-supplement-1.pdf]

bioRxiv preprint doi: https://doi.org/10.1101/2024.12.17.628970; this version posted December 17, 2024. The copyright holder for this preprint (which was not certified by peer review) is the author/funder, who has granted bioRxiv a license to display the preprint in perpetuity. It is made available under aCC-BY 4.0 International license.

Supplemental Figures

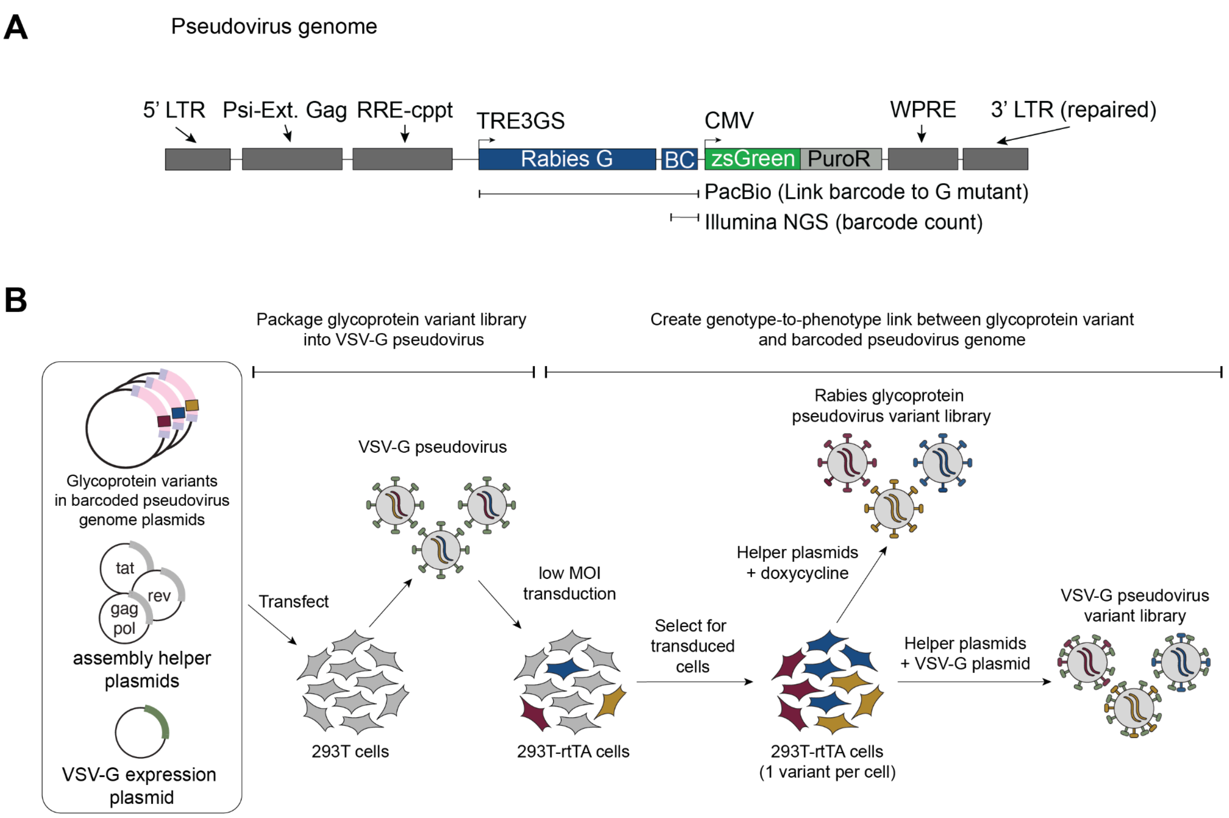

C

Library	Number of barcodes	% of all amino acid mutants in library
A	83706	99.7
B	89327	99.6

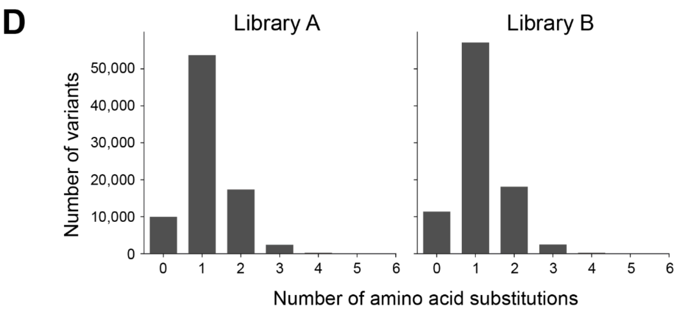

Figure S1. Pseudovirus deep mutational scanning of rabies G (Related to figure 1)

(A) Schematic of lentiviral genome used to produce genotype-phenotype linked pseudovirus libraries. The genome encodes a dox-inducible rabies G mutant gene followed by a stop codon and 16 random nucleotides that function as a barcode. PacBio sequencing is used to link each barcode to a corresponding G mutant. In subsequent experiments, short-read sequencing of barcodes is used to measure cell entry for G mutants. There is a separate transcriptional cassette that constitutively expresses ZsGreen and a puromycin resistance gene. The 3′ LTR is repaired to enable reactivation

bioRxiv preprint doi: https://doi.org/10.1101/2024.12.17.628970; this version posted December 17, 2024. The copyright holder for this preprint (which was not certified by peer review) is the author/funder, who has granted bioRxiv a license to display the preprint in perpetuity. It is made available under aCC-BY 4.0 International license.

of integrated proviruses. The pseudovirus genome employed in this study contains an extended Gag sequence (denoted as "Ext. Gag"), which may improve genome packaging into virions.

(B) Workflow for generating genotype-to-phenotype linked pseudoviruses. We produce VSV-G pseudovirus particles with lentiviral genomes that encode the rabies G mutants and their barcodes. VSV-G pseudovirus are transduced into a 293T-rtTA expressing cell-line at a low MOI to ensure one genome integration per cell. Cells with integrated proviruses selected using puromycin. Library pseudoviruses are produced by transfecting only lentiviral helper plasmids (gag-pol, tat, and rev) and adding doxycycline to induce rabies G expression. VSV-G pseudovirus is simultaneously produced as a control to measure library composition for cell entry measurements by transfecting a VSV-G expression plasmid alongside the helper plasmids.

(C) Number of barcoded variants and percentage of all 8,227 targeted amino acid mutations represented in each of the two replicate G pseudovirus libraries.

(D) Distribution of number of amino acid mutations per G variant in each pseudovirus library. Most sequences contain only one mutation, but some contain no or multiple mutations.

bioRxiv preprint doi: https://doi.org/10.1101/2024.12.17.628970; this version posted December 17, 2024. The copyright holder for this preprint (which was not certified by peer review) is the author/funder, who has granted bioRxiv a license to display the preprint in perpetuity. It is made available under aCC-BY 4.0 International license.

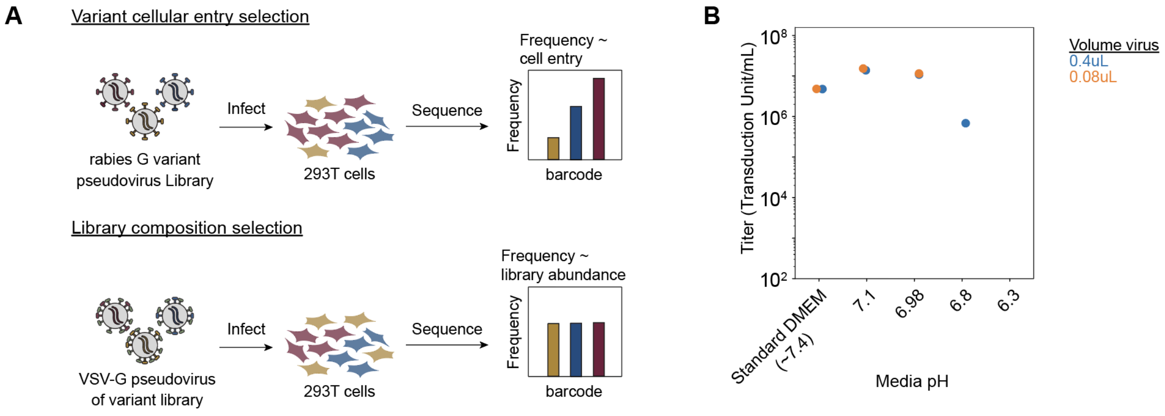

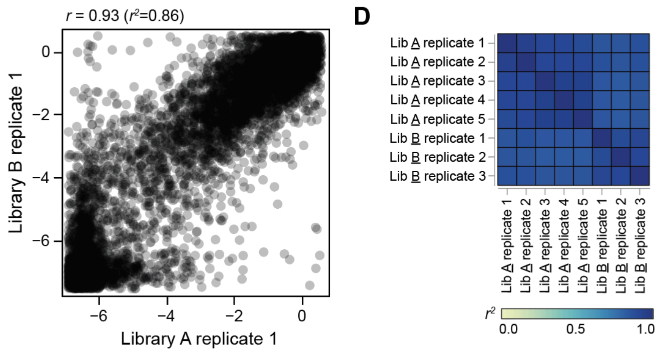

E

Mutant	Effect (cell-based syncytia)	DMS cell entry	Source
D211A	No fusogenic activity	-7.10	Yang, et al (2020).
H261A	No fusogenic activity	-6.14	Yang, et al (2020).
H261L	No fusogenic activity	-6.78	Ng, et al (2022).
D266A	Fusogenic activity	-2.95	Yang, et al (2020).
E269A	Fusogenic activity	-1.31	Yang, et al (2020).
H270P	No fusogenic activity	-6.75	Ng, et al (2022).

Figure S2. Measuring effects of rabies G mutations on cell entry (Related to Figure 1)

(A) Workflow for measuring effects of mutations on cell entry. 293T cells are infected either with pseudovirus expressing only the rabies G mutants, or pseudovirus also expressing VSV-G. After infection, lentiviral genomes are isolated from infected cells and sequenced to identify the fraction of each variant that is able to infect cells. When the virions express only the rabies G mutant, only functional mutants will infect cells. But when VSV-G is also expressed, then all variants can infect cells. The cell entry score for each variant is quantified as the log of its frequency relative to unmutated rabies G in the condition expressing only rabies G versus the condition also expressing VSV-G.

(B) The rabies G pseudovirus infections were performed using media with a pH adjusted to 7.1 as that yielded higher titers. Shown are the titers of a stock of pseudovirus expressing unmutated rabies G in media equilibrated to various pHs.

(C) Correlation of the effects of G protein mutations on cell entry measured in a single experimental replicate with each of the two duplicate pseudovirus libraries..

(D) Overall we performed five repeats of the cell entry measurements using library A and three repeats using library B. The heatmap shows the correlation of the mutation effects on cell entry between each pair of measurements. Throughout this paper, we use the median measurement across replicates for each mutation effect.

bioRxiv preprint doi: https://doi.org/10.1101/2024.12.17.628970; this version posted December 17, 2024. The copyright holder for this preprint (which was not certified by peer review) is the author/funder, who has granted bioRxiv a license to display the preprint in perpetuity. It is made available under aCC-BY 4.0 International license.

(E) Summary of fusogenic activity for rabies G mutants assayed by cell-based syncytia assays as compared to deep mutational scan (DMS) cell entry scores. More negative deep mutational scanning scores correspond to reduced cell entry activity, with scores ≤ -5 indicating mutations that are as deleterious as most stop codons.

bioRxiv preprint doi: https://doi.org/10.1101/2024.12.17.628970; this version posted December 17, 2024. The copyright holder for this preprint (which was not certified by peer review) is the author/funder, who has granted bioRxiv a license to display the preprint in perpetuity. It is made available under aCC-BY 4.0 International license.

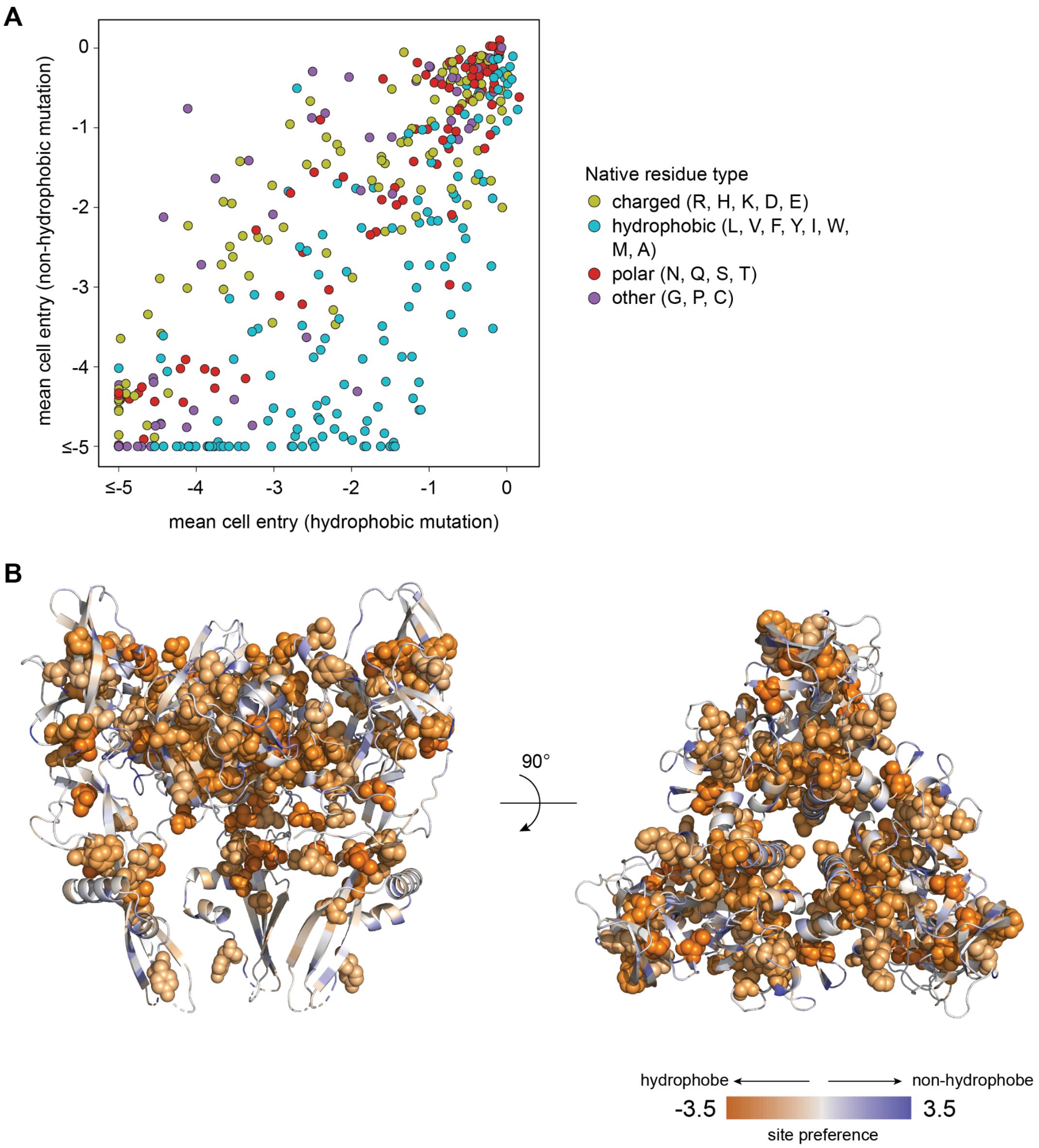

Figure S3. Constraint on hydrophobic residues in rabies G (Related to Figure 2).

(A) Mean effect on cell entry for all mutations to non-hydrophobic amino acids versus the mean effect of all mutations to hydrophobic amino acids. Each point is a site, and is colored by whether the parental amino acid in G at that site is charged, hydrophobic, polar, or other as delineated in the key to the right of the plot. For the calculation of the mean effects, both charged and polar amino acids are grouped into the non-hydrophobic category, and the other category (G, P, and C) are excluded from the mean-effect calculations.

(B) Pre-fusion rabies G structure colored by the preference of each site for hydrophobic versus non-hydrohobic amino acids. This preference score is calculated by subtracting

bioRxiv preprint doi: https://doi.org/10.1101/2024.12.17.628970; this version posted December 17, 2024. The copyright holder for this preprint (which was not certified by peer review) is the author/funder, who has granted bioRxiv a license to display the preprint in perpetuity. It is made available under aCC-BY 4.0 International license.

the mean effect of all mutations to hydrophobic amino acids from the mean effect of all mutations to non-hydrophobic amino acids, so that negative values indicate a preference for hydrophobic residues. The structure is colored so that sites that prefer hydrophobic amino acids are colored orange, and ones that prefer non-hydrophobic amino acids are colored blue. The structure is shown as a cartoon with sites with a hydrophobicity preference more than one standard deviation below the mean of all sites shown in spheres. As expected, sites with hydrophobic preferences tend to be on the core rather than the surface of the protein.

bioRxiv preprint doi: https://doi.org/10.1101/2024.12.17.628970; this version posted December 17, 2024. The copyright holder for this preprint (which was not certified by peer review) is the author/funder, who has granted bioRxiv a license to display the preprint in perpetuity. It is made available under aCC-BY 4.0 International license.

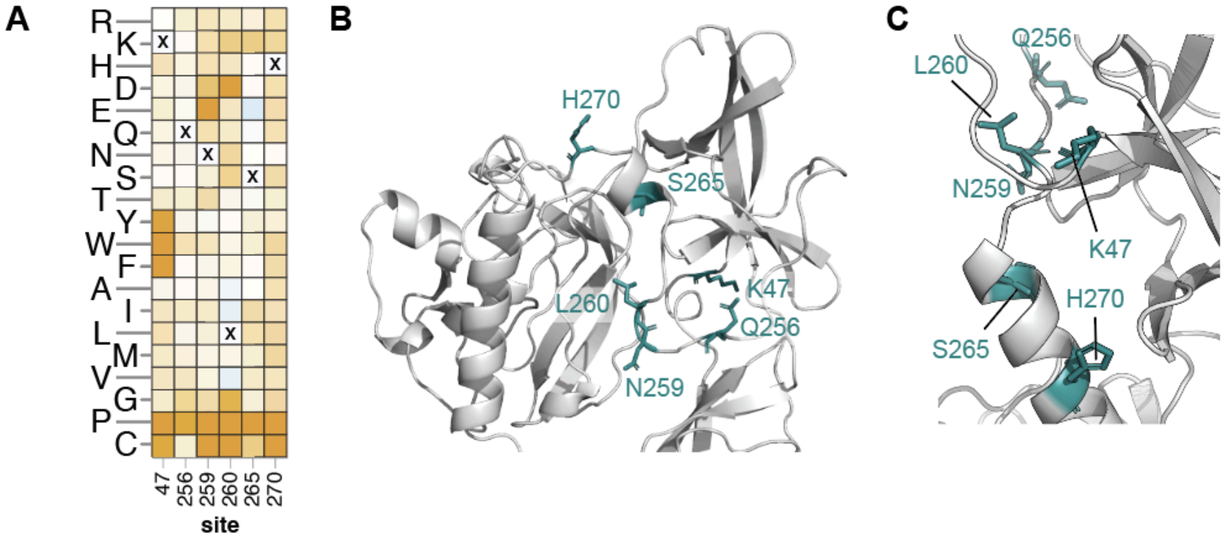

Figure S4. Sites where proline mutations are especially deleterious to cell entry (Related to Figure 2).

(A) Effects of all mutations on cell entry (color scale as in Figure 1C) for a subset of sites where mutations to proline are highly deleterious but mutations to many other amino acids are well tolerated.

(B) Zoomed in view of a single protomer of the pre-fusion trimer with the sites where proline mutations are highly deleterious shown in teal.

(C) Zoomed in view of a single protomer in the extended intermediate conformation with the sites where proline mutations are highly deleterious shown in teal.

bioRxiv preprint doi: https://doi.org/10.1101/2024.12.17.628970; this version posted December 17, 2024. The copyright holder for this preprint (which was not certified by peer review) is the author/funder, who has granted bioRxiv a license to display the preprint in perpetuity. It is made available under aCC-BY 4.0 International license.

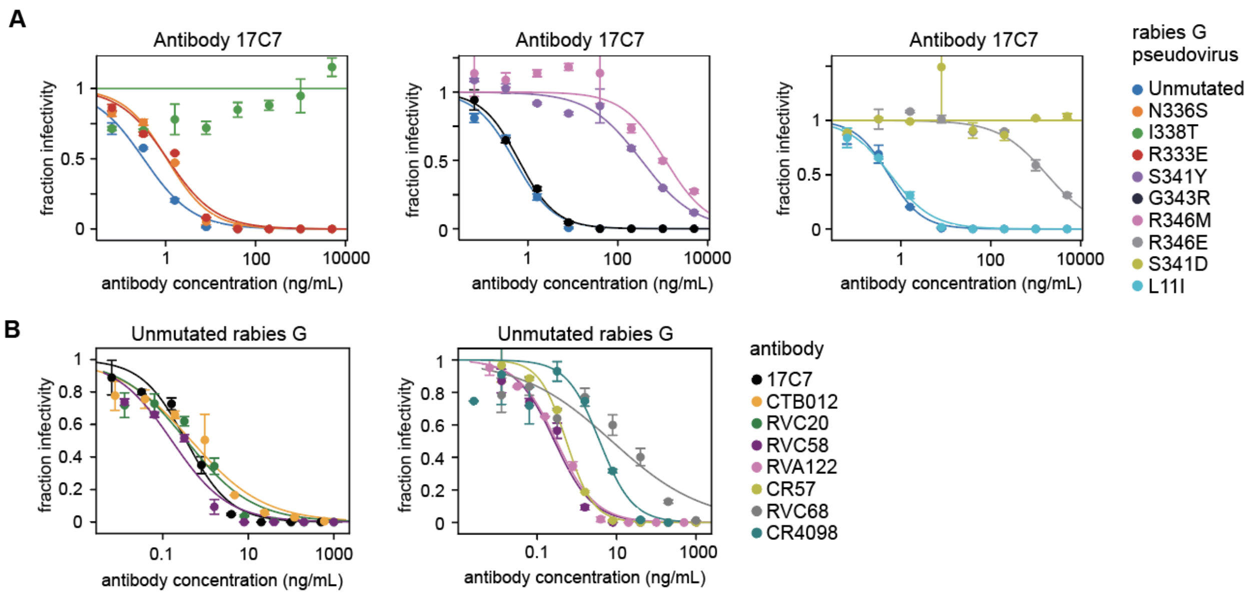

Figure S5. Neutralization curves (Related to Table 1 and Figures 3 and 4).

(A) Neutralization curves for validation pseudovirus assays for nine rabies Pasteur strain G mutants against antibody 17C7. The neutralization assays for all nine mutants were split across three plates, each of which is shown in a separate panel. Unmutated rabies G pseudovirus was run on each plate as a control for plate-dependent variation. Each measurement point represents the mean ± standard error of two technical replicates. A Hill curve was fit to each mutant (solid line) to calculate the IC50.

(B) Neutralization curves for unmutated Pasteur strain rabies G against eight antibodies studied. The neutralization assays were split across different experiments, each of which is shown in a separate panel. RVC58 was run on both plates as an internal control for variation.

bioRxiv preprint doi: https://doi.org/10.1101/2024.12.17.628970; this version posted December 17, 2024. The copyright holder for this preprint (which was not certified by peer review) is the author/funder, who has granted bioRxiv a license to display the preprint in perpetuity. It is made available under aCC-BY 4.0 International license.

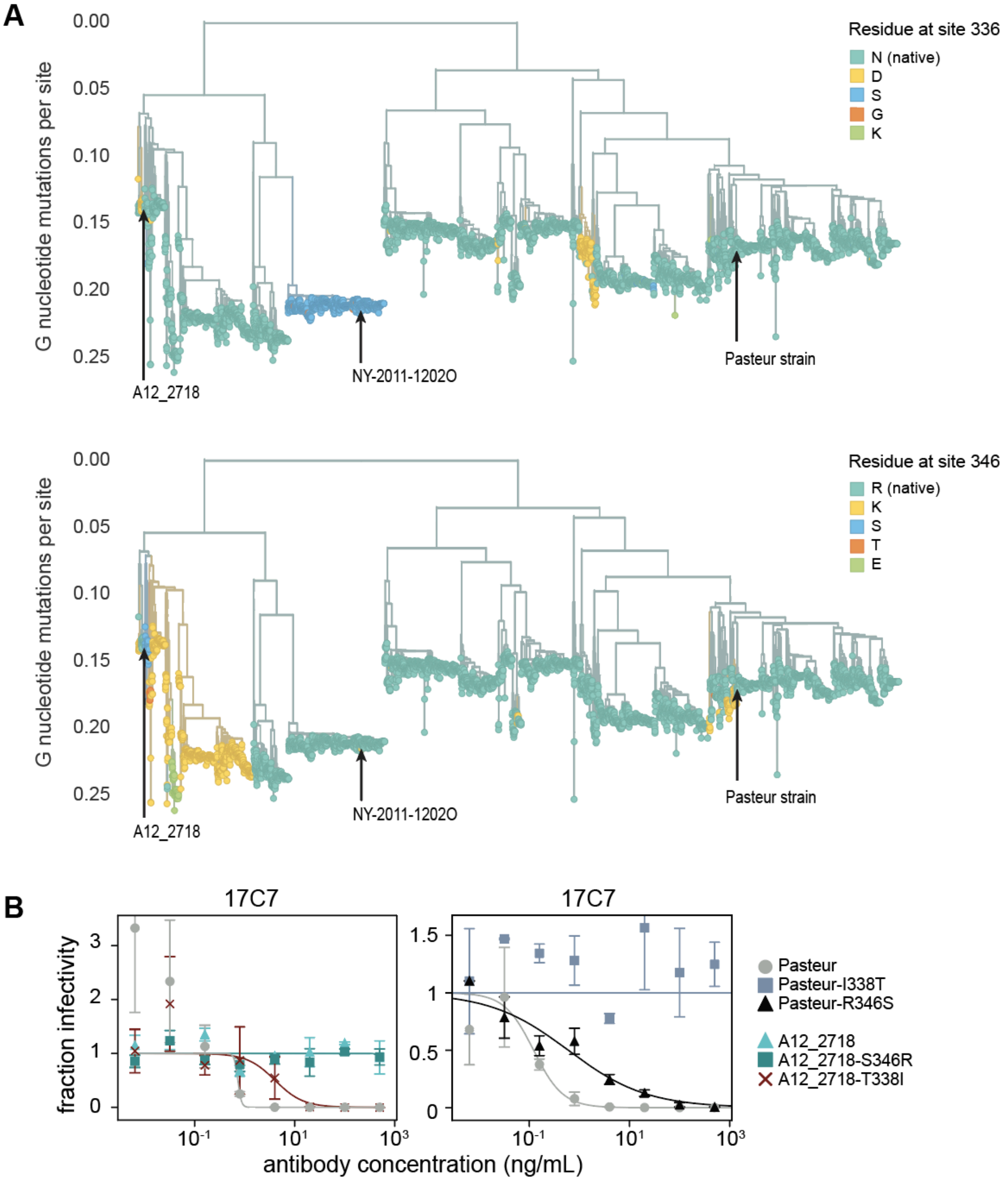

Figure S6. Escape mutations in circulating rabies G sequences (Related to Figure 6).

(A) Phylogenetic trees of all publicly available rabies G sequences colored by the amino-acid identity at sites 336 and 346. Strains tested in the validation assays in Fig 5D are labeled. See https://nextstrain.org/groups/jbloomlab/dms/rabies-G for interactive trees that enable you to color the tree by the amino-acid identity at any site in G.

(B) Neutralization curves of pseudovirus expressing single mutants in rabies G for both the Pasteur and A12_2718 strain backgrounds against antibody 17C7. Each panel represents assays run on a different plate, and points are the mean and standard error of technical

bioRxiv preprint doi: https://doi.org/10.1101/2024.12.17.628970; this version posted December 17, 2024. The copyright holder for this preprint (which was not certified by peer review) is the author/funder, who has granted bioRxiv a license to display the preprint in perpetuity. It is made available under aCC-BY 4.0 International license.

duplicates. Note that as indicated in Fig 5C, I338T and R346S are the top two escape mutations found in strain A12_2718 relative to the Pasteur strain.
